# PIWI-interacting RNAs: new biomarkers for diagnosis and treatment of breast cancer

**DOI:** 10.1186/s13578-020-00403-5

**Published:** 2020-03-23

**Authors:** Parisa Maleki Dana, Mohammad Ali Mansournia, Seyyed Mehdi Mirhashemi

**Affiliations:** 1 Research Center for Biochemistry and Nutrition in Metabolic Diseases, Kashan University of Medical Sciences, Kashan, I.R. of Iran; 2 Department of Epidemiology and Biostatistics, School of Public Health, Tehran University of Medical Sciences, Tehran, Iran; 3 Metabolic Diseases Research Center, Research Institute for Prevention of Non-Communicable Diseases, Qazvin University of Medical Sciences, Qazvin, Iran

**Keywords:** piRNA, Breast cancer, Epigenetic regulations

## Abstract

Cancer is one of the most important reasons of mortality in the world. However, there are several therapeutic platforms to treat patients who suffering from cancer common treatments such as surgery, chemotherapy and etc. The current therapeutic approaches are related to some limitations. Hence, more understanding about molecular mechanisms that involved in cancer particularly in breast cancer pathogenesis, could contribute to provide better therapeutic platforms. Recently, non-coding RNAs such as microRNAs have attracted researchers’ attention in the field of cancer due to their functions in gene expression’s regulation and functional interactions with other molecules. Interestingly, great advances in next-generation sequencing lead to considering other roles for another non-coding RNAs subgroup called PIWI-interacting RNAs (piRNAs) in addition to their functions in the germline. Novel studies investigated the role of piRNAs in several cancers including lung cancer, hepatocellular carcinoma, gastric cancer, multiple myeloma and colorectal cancer. Hopefully, based on new findings, piRNAs may be a potential biomarker which can be used as a tool to diagnose or treat breast cancer. Thus, this review aimed to discuss the role of piRNAs in breast cancer progression and metastasis as well as its molecular mechanisms.

## Background

Breast disorders are very important disorders among women [1]. Among them, Breast cancer is the most common malignancy among women and it is the principle cause of women’s death all over the world. In spite of great progress in the field of cancer, breast cancer is still a serious health problem among women which has complicated properties heterogeneously and shows a number of biological and clinical manifestations [2–4]. Breast cancer has been categorized into five groups based on expression patterns of three receptors: the estrogen receptor, progesterone receptor and human epidermal growth factor receptor. This categorization was a helpful way in order to predict the outcome and choose the best treatment option [5, 6]. Mutations in BRCA1 and BRCA2 are some examples of genomic instabilities which are the most prominent cause of breast cancer [7–9]. Several studies indicated that a variety of techniques could be used in the treatment of different cancers such as breast cancer (i.e., cell-, gene-, and nanotechnology-based therapies) [10–17]. Despite emerging new and effective therapeutic platforms in the treatment of breast cancer, new approaches are needed [18, 19]. In this regard, it seems that more understanding of cellular and molecular pathways involved in breast cancer pathogenesis could contribute to the development of new therapies [18].

In the past, scientists called 98% non-protein coding human genome as “junk” DNA. These DNAs produce RNAs which are not translated into proteins. This group is known as non-coding RNAs (non-encoding RNAs) which has two main subgroups: regulatory non-coding RNAs and housekeeping non-coding RNAs. The regulatory non-coding RNAs are also sorted into two subdivisions by their length: short chain non-coding RNAs and long chain non-coding RNAs. Short chain non-coding RNAs include small interfering RNAs (siRNAs), microRNAs (miRNAs), and piwi-interacting RNAs (piRNAs) [20]. Moreover, long chain non-coding RNAs are known as long non-coding RNAs (lncRNAs) and circularRNAs [20, 21]. Recently, it has been revealed that non-coding RNAs have considerable roles in modifying epigenetics and regulating gene-level and chromosome-level expressions in order to cell differentiation controlling [22–24]. piRNAs are RNA molecules with the estimated size of 26–31 nt binding to Piwi proteins [25, 26]. The Piwi protein with the polycomb group proteins (PcGs) together bind to genomic PcG response elements in order to act as a regulatory factor. Therefore, piRNAs have a regulatory function in the body [27]. PiRNAs are classified into 3 group’s base on their origin: transposon-derived piRNA, lncRNA-derived piRNAs, and mRNA-derived piRNAs. Besides, there are two sub-clusters for piRNAs; one of them acts in premeiotic germ cells (pre-pachytene piRNAs) and another one function in meiosis and haploid spermatid stage (pachytene piRNAs). The molecular features of both of these piRNAs are the same but pre-pachytene piRNAs cluster is totally different and has repetitive sequence elements [28]. piRNA-induced silencing complexes are produced by binding of piRNAs to PIWI proteins which generate a germline-specific member of the Argonaute family acting in silencing genes occurred by small RNAs [29].

piRNA-induced silencing complexes function by suppressing transposons during transcription and post-transcription leading to protection of the integrity of the germline genome. Moreover, regulation of cellular genes is another role of piRNAs in a variety of organisms [30–32]. PIWI–piRNA pathway acts both in inside and outside the germline. Additionally, these RNAs can recognize “self” and “nonself” genes and regulate the latter [33]. PIWI protein genes also exist in Drosophila and mouse; (ago3, aubergine (aub), and piwi), (MIWI, MIWI2, and MILI) are their distinct genes respectively. It is observed in these eukaryotes that PIWI proteins are involved in transposon silencing, undeniable function in gonadal development, male and female fertility, DNA methylation and etc. [34]. In addition to gonadal functions of PIWIs, they play roles in somatic tissues, especially stem cells. Up to now, four human PIWIs have been identified including HIWI (also known as PIWIL1), HILI (also known as PIWIL2), HIWI2 (also known as PIWIL4) and HIWI3 (also known as PIWIL3) [31]. Recent findings investigated the role of piRNAs in several cancers including lung cancer [35], hepatocellular carcinoma [36], gastric cancer [37], multiple myeloma [38] and colorectal cancer [39]. Hopefully, based on new research, piRNAs may be potential biomarkers which can be used as a tool to diagnose or treat breast cancer. Thus, this review aimed to discuss the role of piRNAs in breast cancer progression and metastasis as well as its molecular mechanisms.

### PiRNA biosynthesis

While the transcription of transposon-derived piRNAs occurs from both genomic strands, sense and antisense piRNAs can be generated. While the origin of mRNA-derived piRNAs is the 3′ untranslated regions of mRNAs, the lncRNAs-derived piRNAs originate from the entire lncRNA transcript [40]. Unlike miRNAs and siRNAs which are dependent on RNAse III Dicer for their maturation, piRNA biogenesis is a Dicer-independent process [41, 42]. Additional post-transcriptional processes are necessary for the full maturation of piRNAs precursors. The primary synthesis and ‘ping-pong’ amplification are the two mechanisms which are used after the transcription in order to produce mature piRNAs [43]. In the simplest scenario, in gonad somatic cells of *Drosophila*, riboendonuclease Zucchini cleaves the primary transcript at first. After the incorporation of 3′ fragment in PIWI proteins, it is trimmed to its final length by a 3′ to 5′ exonuclease. The enzyme Hen1 methylate the 2′ hydroxy group of the 3′ end. Meanwhile, 5′ end residue of incorporated piRNA in PIWI has a strong bias for the residues of uridine. Then, piRNAs bind PIWI proteins and form piRNA/PIWI complexes [44]. Finally, these complexes migrate to the nucleus where they can mobilize the silencer machinery and block their target gene transcription. Therefore, heterochromatin which is transcriptionally silent will be established through the piRNA-mediated recruitment of histone methyltransferases [45]. In the cytoplasm, the ping-pong mechanism amplifies the accumulation of piRNAs after the production of primary piRNA [44]. Unlike the primary synthesis which is related to PIWI proteins, in this process, piRNA/Ago3 or piRNA/Aub complexes are formed. An RNA sequence will be generated by the piRNA/Ago3 complex which acts as a substrate used for producing new piRNA and the generated piRNA can load an Aub protein [46]. In fact, through an amplifying mechanism, the products of piRNAs result in the formation of substrate for other functional piRNAs [47]. While studies indicated that ‘ping-pong’ mechanism exists in *zebrafish*, *D. melanogaster* and primitive animals, findings reveal that the biogenesis of piRNAs in mice is not dependent on the ‘ping-pong’ cycle [47, 48].

As a mechanism which is firstly explained in ovarian germline of fly, the endonucleolytic action of cytosolic PIWI leads to the formation of two cleavage fragments. Then, new mature piRNA is produced through the participation of the downstream fragment in biogenesis pathway of piRNA. Noteworthy, PIWI slicing generates 5′ end of secondary piRNAs [44, 49]. Moreover, it is reported that the downstream cleavage fragment can be the origin of an extensive biogenesis of piRNAs [50–52]. In inchworming process, the fragment produces other non-overlapping piRNAs in a direction occurring from 5′ to 3′ [51]. Inchworming-produced primary piRNAs load into PIWI proteins of fly nucleus. Two PIWI proteins of mouse have been showed to have a similar association. In diverse species, biogenesis of piRNA happens near the mitochondrial surface, and different mitochondrial membrane-anchored factors are involved [53]. In mice, two cytoplasmic PIWI proteins including MILI, and MIWI, receive processed pachytene piRNAs at intermitochodrial cement (IMC) [53].In fact, what ensures that transposons remain silenced is the relation between cytoplasmic MILI and nuclear MIWI2 in the mouse embryonic male germline [54]. Moreover, the slicer activity of MILI has been reported to be essential in the production of MIWI2-associated piRNAs [55]. In a study of Yang et al. [56], it is shown that transcript endonucleolytic slicing which is done by cytosolic PIWI proteins of mouse, MILI, stimulates its 5′ → 3′ processing. This event eventually leads to the production of non-overlapping fragments which are new piRNAs. Then, new piRNAs accumulate in nuclear MIWI2 and cytosolic MILI. Exonuclease domain-containing 1 (EXD1) is found to be the partner of TDRD12 which is a factor for the biogenesis of MIWI2 piRNA. Despite the inactivity of EXD1 as a nuclease, this factor plays its role in PET (PIWI-EXD1-Tdrd12) complex as an adaptor of RNA. Altogether, this study concluded that PIWI stimulates the piRNA biogenesis and EXD1 enhances the production of piRNAs and ensures the effective entry of small RNAs to the PIWI proteins of nucleus [56].

### PiRNAs and cancer: molecular mechanisms

In addition to PIWI proteins, piRNAs have significant roles in the carcinogenesis. Reproductive tissues express a great number of piRNAs. PiRNAs are also expressed in brain tissue as well as exosomes which are derived from the human plasma [57, 58]. While miRNAs serve as the regulator of post-transcriptional activities, piRNAs are considered to act as the epigenetic regulator which is involved in angiogenesis, invasiveness, growth and metastasis of tumors [59, 60]. Up to now, several studies concerned with the role of piRNAs in different processes which are involved in cancer.

PiR-651 has been shown to play a role in carcinogenesis through affecting apoptosis, proliferation, and migration. This piRNA is concluded to be overexpressed in several cancer cell lines such as lung, colon, gastric, breast cancer, hepatic carcinoma and mesothelioma. Moreover, it is found that the inhibitor of piR-651 can make cancer cells stop in G2/M phase. Indeed, due to the impacts of the piRNA pathway on the balance of cell division and self-renewal, any interruption in this pathway may make an effect on the progression of cancer. There are several studies indicating that the expression of piRNAs is decreased in multiple cancers resulting in the proliferation of cancer cells and progression of tumors [61]. As the piR-55490 is downregulated in lung cancer, research indicates that the proliferation of lung cancer cells can be reduced through the recovery of this piRNA [62]. Similar to what miRNAs do, piR-55490 decreases the decay of mTOR mRNA through binding to its 3′ UTR; thus, inhibiting Akt/mTOR signaling in lung cancer cells. In other words, piRNA targets oncogenic mRNA and leads to the suppression of tumor cells [62].

Novel research which is concerned with somatic roles of piRNAs has revealed that piRNAs may be involved in the regulation of gene expression by histone modification and DNA methylation [31, 63–65]. Meanwhile, DNA hypomethylation, histones hypoacetylation and gene-specific DNA hypermethylation are general epigenetic changes occurred in cancer leading to R-ras and cyclin D2 oncogenes activation as well as silencing RB1 and p16 tumor suppressor genes [59, 66]. PIWIL4 (MIWI2) embedded in the nucleus leads to the methylation of retrotransposons promoter sequences and suppress them through methylation of de novo DNA in male mice before birth. Furthermore, by mutating MIWI2 and MIWI, retrotransposon promoters methylation will be lost in the mouse testes [67, 68]. In several types of cancer such as lung, breast, colorectal, and stomach cancer, piR-1245 is reported to be overexpressed and considered to be involved in the carcinogenesis [69]. In colorectal cancer, this piRNA has been observed to regulate the progression of the tumor by targeting ATF3, BTG1, DUSP1, FAS, NFKBIA, UPP1, SESN2, TP53INP1 and MDX1 and associated with poor differentiation and metastasis [70]. While DNMT3L/PIWIL2/TDRD1 complex can cause DNA methylation loss at transposons (LINE1 and IA), data has shown that in primary tumors of the testicle, transcriptional silencing is linked to the methylation of 5′ end promoter CpG in different genes including PIWIL1, PIWIL2, PIWIL4 and TDRD1 [71].

There is a theory explaining that piRNAs and PIWI proteins may lead to a characteristic found in cancers, aberrantly “stem-like” state. This stem-like state is occurred by the of PIWI-piRNA complexes that lead to genomic silencing following the aberrant methylation of DNA. Moreover, cancer cells which are similar to stem cells have shown to gain metastatic features as well as undergoing epithelial-mesenchymal transition. Despite the fact that epigenetic aberrancies are important tumor tissues properties, they are not well-understood yet. Emerging evidence collected from different types of tumors has suggested that cancers reflect the epigenetic state of adult progenitor cells [72]. Moreover, inhibiting mechanisms affecting the hypermethylation of DNA is similar to progenitor cells [73]. However, in progenitor cells, DNA methylation is responsible for silencing the differentiation genes, promoter site of tumor suppressor genes are the regions where the hypo-methylation occurred in cancer [73]. In stem cells, differentiation genes are silenced by EZH2 methyltransferase which mediates repressive histone 3 lysine 27 tri-methylation mark [74]. Furthermore, overexpression of EZH2 is observed in many cancers such as breast and prostate cancer which are associated with tumor aggressiveness [75]. These findings together may confirm that cancers have an aberrantly “stem-like” epigenetic state [76]. Findings have shown that piRNAs are considered to take part in different mechanisms involved in cancer including tumor cells proliferation, apoptosis, cancer spreading, and invasion and possibly be a potential biomarker for prognosis and diagnosis of cancer. The apoptosis-inducing role of piRNAs and their ability to prevent cell proliferation has been investigated in various tumors such as breast cancer, bladder cancer, multiple myeloma, gastric cancer, glioma and etc. The inhibitory function of piRNA on metastasis and invasion has been also shown in breast cancer, clear cell renal cell carcinoma, gastric cancer, and hepatocellular carcinoma [77].

### PiRNAs and breast cancer

For the first time, Fu et al. [78] found that epigenetic functions occurring by piRNAs are able to make an effect on cancer-related pathways which are also involved in human breast cancer. Although their findings were restricted to one cell line, it is suggested to be capable to affect various genes methylation in MCF7. Indeed, the results showed that certain piRNAs, like miRNAs associated with cancer, probably target multiple genes thus any irruption in their function may lead to serious consequences such as several downstream of cancer-related mechanisms. Based on evidence gathered from this study, piR-021285 can modulate the invasiveness of human breast cancer through methylation of the pro-invasive *ARHGAP11A* gene at CpG site which is existed in the region of 5′ UTR/first exon. Consequentially, the expression of pro-apoptotic mRNA will be decreased and apoptosis will be inhibited.

In a recent study, it is observed that piRNA-36,712 level in breast tumor tissues was significantly lower than in healthy tissues. In addition, low levels of this piRNA were related to poor outcome in breast cancer patients. Several studies showed that a retroprocessed pseudogene of *SEPW1 “SEPW1P”* produces RNAs which have interaction with piRNA-36,712. Thus, the competition of *SEPW1* mRNA with *SEPW1P* RNA for microRNA-7 and microRNA-324 leads to SEPW1 suppression. Moreover, downregulation of piRNA-36,712 resulting in an increase in SEPW1 expression may inhibit P53 which contributes to Slug upregulation and P21 and E-cadherin levels reduction. Altogether, these alterations cause to promote the proliferation of cancer cells, invasion and migration. Besides, piRNA-36,712 can amplify the effects of chemotherapeutic agents, paclitaxel and doxorubicin in breast cancer. These findings implicate that piRNA-36,712 is a new tumor suppressor which can be used in order to provide a prognosis and treat breast cancer patient [79].

In another study, the role of piRNAs in human breast cancer cells was examined. Using statistical analysis (Wilcoxon Mann–Whitney test) revealed a tumor-specific pattern containing 8 piRNAs which are expressed differentially. Five of these piRNA expression levels were lower in breast cancer tissues including DQ596670, DQ598183, DQ597341, DQ598252 and DQ596311 and three of them had higher expression levels in comparison with normal tissues, including DQ598677, DQ597960 and DQ570994. Recent research implicated that piRNAs have a role similar to miRNAs as they enhance to the formation of specific RNA silencing complexes (pi-RISC) promoting suppression of RNAs through an incomplete base-pairing between two of them [80, 81]. In Rizzo et al. [82] study applying stringent thermodynamic parameters and binding energy thresholds, it is indicated that each piRNA differentially expressed in cancer tissues was complementary to 23 to 383 RNAs comprising: mRNAs, pseudogene transcripts and long ncRNAs. These mRNAs probably targeted by piRNAs encode proteins which mainly have functions in breast cancer important cellular processes including cell-to-cell signaling and interaction, cell death and survival, cell cycle, and DNA replication and repair. Hu et al. [83] evaluated piRNA expression in tumor tissues and their non-tumoral counterparts by real-time PCR. The results indicated that four out of six piRNAs were up-regulated in breast cancer tissue including piR-4987, piR-20365, piR-20485, and piR-20582. Also, upregulation of piR-4987 expression was linked with lymph node positivity resulting in poorer outcome in breast cancer patients.

The most prominent prognostic indicator in breast cancer is axillary lymph node status which is also used to indicate the capability of the tumor to metastasis [84]. Based on this information, piR-4987 is a key player in progression, invasion and tumor spreading, thus piR-4987 overexpression leads to more aggressive breast cancer. Due to piRNAs role which is silencing transposons leading to genetic mutation and being associated with cancer, piRNAs may affect cancer by regulation of transposons. In addition, piRNAs probably have a regulatory effect on genes responsible for coding proteins. Thus, piRNA in cancer can regulate both oncogenes and anti-oncogenes. Another investigation on piR-651 revealed that its upregulation occurs in multiple cancers including breast cancer. This finding suggested that piR-651 may be an oncogene which was confirmed in gastric cancer. Guo et al. [39] used piR-651 inhibitor which leads to suppressing of gastric cancer cell growth. Their study implied that any disruption in the balance of this piRNA may result in a significant effect on tumor progression. The piR-932 expression has been also investigated in breast cancer. It is declared that piR-932 is significantly expressed higher in breast tumor tissues than in normal tissues. Furthermore, it can create immune complexes by immunoprecipitation with the help of PIWIL2. Also, in PIWIL2 + breast cancer stem cells, due to the methylation of Latexin promotor region (CpG island), Latexin expression levels are significantly lower than normal. These findings introduce a potential target which is the combination of piR-932 and PIWIL2. This complex, as a positive regulator promotes Latexin methylation and can be used in order to block the metastasis in breast cancer [85].

### PIWI proteins and breast cancer

Similarly, PIWI proteins also have significant roles in different cancers such as breast cancer. In spite of extensive research about cancer, only recent articles have discussed PIWI proteins roles in this area [86]. PIWIL4 Gene is one of the upregulated genes in breast cancer which is highly expressed not only in breast cell lines but also in breast cancer samples. Findings have shown that in various cancers, PIWI proteins are expressed aberrantly. For instance, the overexpression of PIWIL2 has been observed in breast cancer [76, 87–92].

Moreover, Wang et al. [86] investigated the three active PIWI genes (PIWIL1, PIWIL2 and PIWIL4) expression. However, this study performed on six different types of breast cancer cell lines and the results showed that PIWIL1 expression level is significantly higher in four cancer cell line than in normal breast cell line. Additionally, PIWIL2 over-expressed in two cancer cell lines. Furthermore, PIWIL4 has remarkably higher expression levels in five cell lines. Another study also found that in 334 out of 1086 breast cases, PIWIL2 increased in breast cancer stem cells. Significantly, PIWIL2 has been linked to age, size of the tumor, histological type, tumor stage and lymph node metastasis. As mentioned earlier, PIWIL2 and piR-932 together can create immune complexes [85].

## Conclusions

**Fig. 1:**
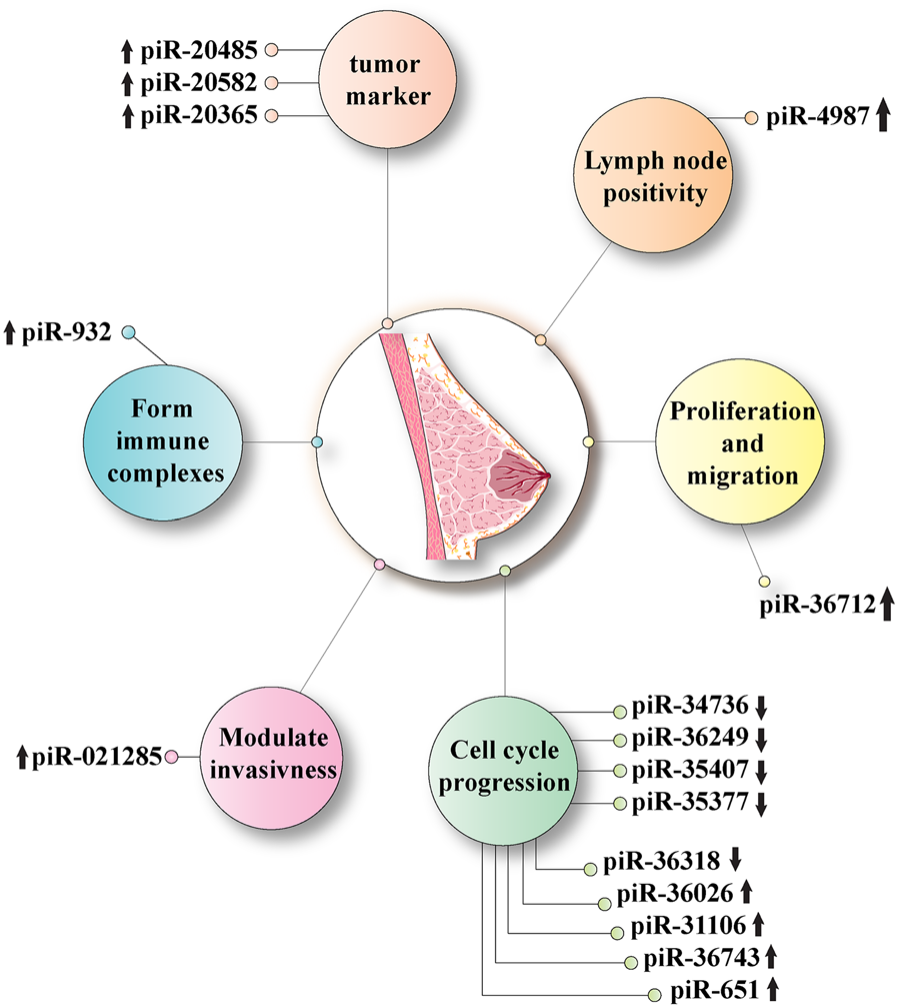
Factors related to PIWI proteins and piRNAs in breast cancer

PiRNAs as a member of non-coding RNAs family have various roles in the germline as well as in somatic cells. It is found that piRNAs act as regulators in many cancers including breast cancer. Several studies have shown that piRNAs are involved in tumor cell’s proliferation, apoptosis, invasion and metastasis. Although the research in this area is in its infancy, several papers implied that piRNAs can be used as biomarkers to early diagnosis of the breast cancer and may be used as a treatment option for this cancer (Fig.1 and Table 1). Expression patterns of piRNAs are different in breast cancer cell lines; in some of them, piRNAs expressions aberrantly increase but, in the others, piRNAs have been seen to be down-regulated or they may be absent totally. Altogether, with further investigations, piRNAs may have a major role in the advancement of breast cancer research while there is a necessity to think beyond the usual breast cancer treatments. Thus, piRNAs may be beneficial targets for diagnosis, treatment or even preventing the metastasis of this cancer.

**Table 1:** Experimental studies that investigated the role of piRNAs in breast cancer

PiRNA	Function	Type of breast cancer or its cell line	Model	Expression	References
PiR-36712	Promotes cancer cell proliferation, invasion and migration	Invasive breast ductal carcinoma	In vitro and In vivo	Underexpressed	[79]
PiR-34736	Progression of cell cycle	Invasive breast ductal carcinoma	In vitro	Underexpressed	[82]
PiR-36249	Progression of cell cycle	Invasive breast ductal carcinoma	In vitro	Underexpressed	[82]
PiR-35407	Progression of cell cycle	Invasive breast ductal carcinoma	In vitro	Underexpressed	[82]
PiR-34377	Progression of cell cycle	Invasive breast ductal carcinoma	In vitro	Underexpressed	[82]
PiR-36318	Progression of cell cycle	Invasive breast ductal carcinoma	In vitro	Underexpressed	[82]
PiR-36026	Progression of cell cycle	Invasive breast ductal carcinoma	In vitro	Overexpressed	[82]
PiR-31106	Progression of cell cycle	Invasive breast ductal carcinoma	In vitro	Overexpressed	[82]
PiR-36743	Progression of cell cycle	Invasive breast ductal carcinoma	In vitro	Overexpressed	[82]
PiR-20582	Tumor marker	Invasive breast ductal carcinoma	In vitro	Overexpressed	[83]
PiR-4987	Lymph node positivity	Invasive breast ductal carcinoma	In vitro	Overexpressed	[83]
PiR-20365	Tumor marker	Invasive breast ductal carcinoma	In vitro	Overexpressed	[83]
PiR-20485	Tumor marker	Invasive breast ductal carcinoma	In vitro	Overexpressed	[83]
PiR-651	Tumor progression	Bcap-37 cell line	In vitro	Overexpressed	[39]
PiR-021285	Modulates Breast cancer invasiveness	MCF7 breast cancer cell lines	In vitro	Overexpressed	[78]
PiR-932	Promotes methylation of Latexin and form immune complexes	Breast cancer stem cells(CSC)	In vitro	Overexpressed	[85]

## Data Availability

Not applicable.
